# Development and evaluation of INT^2^GRATE: a platform for comprehensive assessment of the role of germline variants informed by tumor signature profile in Lynch syndrome

**DOI:** 10.3389/fonc.2023.1284690

**Published:** 2024-01-24

**Authors:** Raymond A. Isidro, Anu Chittenden, McKenzie Walker, Alison Schwartz, Diane R. Koeller, Connor P. Hayes, Busra Unal, Monica Devi Manam, Ryan M. Buehler, Danielle K. Manning, Lynette M. Sholl, Mark S. Redston, Matthew B. Yurgelun, Huma Q. Rana, Judy E. Garber, Arezou A. Ghazani

**Affiliations:** ^1^ Department of Pathology, Brigham and Women’s Hospital, Boston, MA, United States; ^2^ Harvard Medical School, Boston, MA, United States; ^3^ Division of Cancer Genetics and Prevention, Dana-Farber Cancer Institute, Boston, MA, United States; ^4^ Division of Genetics, Brigham and Women’s Hospital, Boston, MA, United States; ^5^ Division of Population Sciences, Dana-Farber Cancer Institute, Boston, MA, United States; ^6^ Department of Medicine, Brigham and Women’s Hospital, Boston, MA, United States

**Keywords:** somatic and germline integration, INT^2^GRATE, tumor signature profile, germline VUS, Lynch syndrome

## Abstract

The presence of variants of uncertain significance (VUS) in DNA mismatch repair (MMR) genes leads to uncertainty in the clinical management of patients being evaluated for Lynch syndrome (LS). Currently, there is no platform to systematically use tumor-derived evidence alongside germline data for the assessment of VUS in relation to LS. We developed INT^2^GRATE (INTegrated INTerpretation of GeRmline And Tumor gEnomes) to leverage information from the tumor genome to inform the potential role of constitutional VUS in MMR genes. INT^2^GRATE platform has two components: a comprehensive evidence-based decision tree that integrates well-established clinico-genomic data from both the tumor and constitutional genomes to help inform the potential relevance of germline VUS in LS; and a web-based user interface (UI). With the INT^2^GRATE decision tree operating in the backend, INT^2^GRATE UI enables the front-end collection of comprehensive clinical genetics and tumor-derived evidence for each VUS to facilitate INT^2^GRATE assessment and data sharing in the publicly accessible ClinVar database. The performance of the INT^2^GRATE decision tree was assessed by qualitative retrospective analysis of genomic data from 5057 cancer patients with MMR alterations which included 52 positive control cases. Of 52 positive control cases with LS and pathogenic MMR alterations, 23 had all the testing parameters for the evaluation by INT^2^GRATE. All these variants were correctly categorized as INT^2^GRATE POSITIVE. The stringent INT^2^GRATE decision tree flagged 29 of positive cases by identifying the absence or unusual presentation of specific evidence, highlighting the conservative INT^2^GRATE logic in favor of a higher degree of confidence in the results. The remaining 99% of cases were correctly categorized as INCONCLUSIVE due to the absence of LS criteria and ≥1 tumor parameters. INT^2^GRATE is an effective platform for clinical and genetics professionals to collect and assess clinical genetics and complimentary tumor-derived information for each germline VUS in suspected LS patients. Furthermore, INT^2^GRATE enables the collation of integrated tumor-derived evidence relevant to germline VUS in LS, and sharing them with a large community, a practice that is needed in precision oncology.

## Introduction

Lynch Syndrome (LS) is a hereditary condition, most commonly associated with colorectal and endometrial cancers (CRC and EC, respectively). With an estimated population prevalence of 1:279 (1), LS is caused by heterozygous germline inactivating alterations in the DNA mismatch repair (MMR) genes *MLH1, MSH2*, *MSH6*, *PMS2*, deletion in *EPCAM*, and other rare epigenetic events (2–4). Although the diagnosis of LS requires the identification of a pathogenic germline MMR gene variant, LS is highly suspected in individuals with certain clinical features and whose tumors exhibit microsatellite instability (MSI) resulting from MMR deficiency (MMRd) (5).

Variant pathogenicity is routinely assessed according to the American College of Medical Genetics (ACMG) guidelines (6), an evidence-based system developed for constitutional Mendelian genetic disorders. This current framework for germline variant assessment does not incorporate tumor-derived evidence and a systematic, evidence-based approach for the joint interpretation of germline and tumor data does not exist. The segregation of germline and somatic variant databases further perpetuates the lack of integration of constitutional and tumor-derived information. We have previously demonstrated the value of the integrated germline and somatic framework in assessing the pathogenicity of germline variants in several cancer syndromes (7–12), and the utility of this integrated approach in the assessment of germline variants of uncertain significance (VUS) in cancer susceptibility genes (7). There is currently a need for objective assessment of VUS in high-risk individuals where germline variants might be actionable, or in other genetic settings where the identification of MMR variants might help preventative approaches.

The aim of the present study is to develop and evaluate INT^2^GRATE (INTegrated INTerpretation of GeRmline And Tumor gEnomes), our evidence-based decision tree for assessing germline VUS in MMR genes in patients in which LS-related CRC or EC is suspected. Using stringent criteria, INT^2^GRATE combines clinical, germline, and tumor-derived data to collect relevant evidence and to inform the potential relevance of germline VUS in LS. The INT^2^GRATE logic is coded in the back end of a web application with a friendly user interface (UI) to facilitate a convenient collation and comprehensive assessment of germline and tumor-derived evidence. The INT^2^GRATE UI also enables the sharing of key tumor and germline variant data in the publicly available ClinVar database by the users. Our goal is to promote integrated germline and tumor-derived assessment and foster data sharing, particularly tumor data associated with germline VUS.

## Methods

### Development of INT^2^GRATE decision tree logic

**Figure 1:**
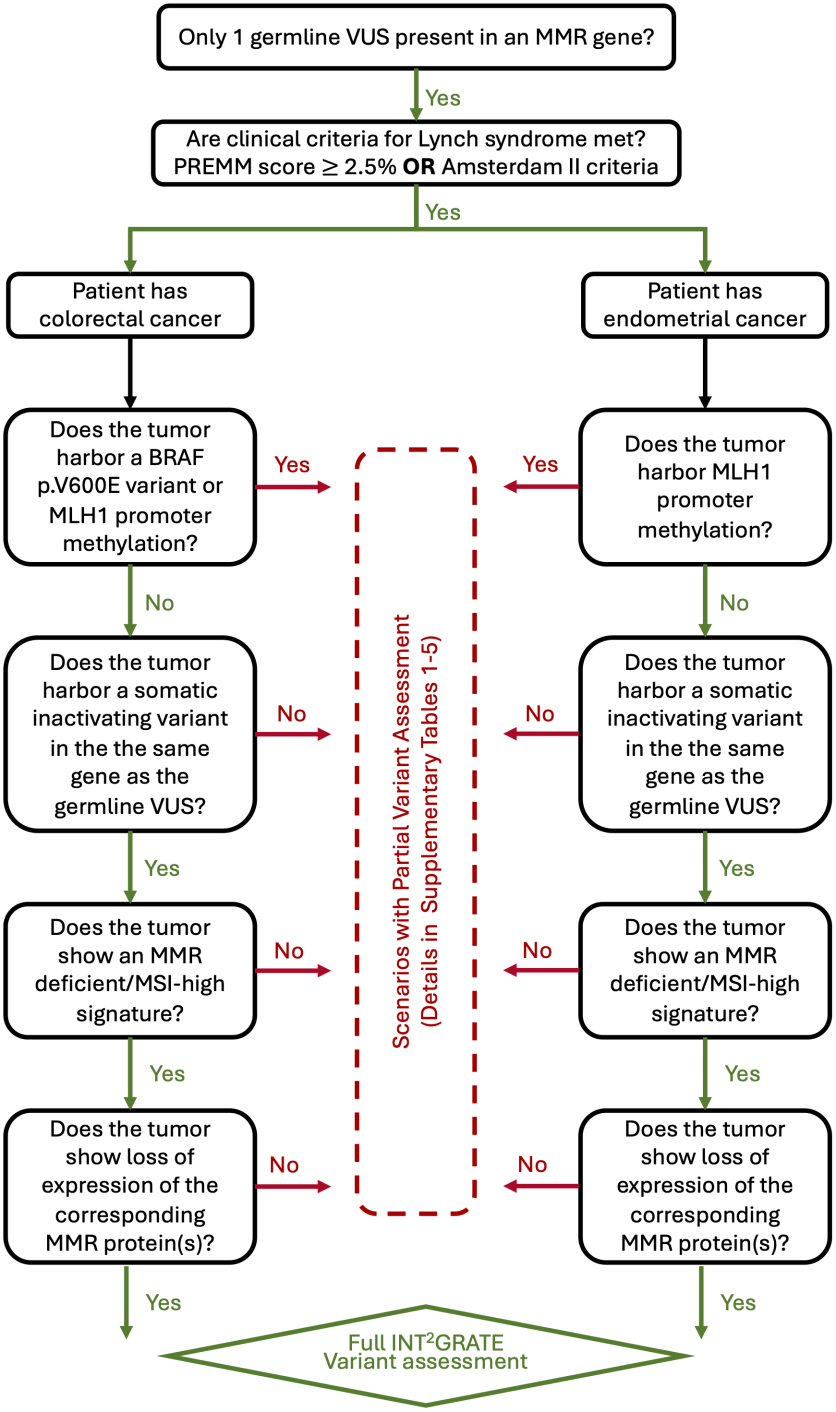
Diagram representing the INT^2^GRATE process for assessing germline variants of uncertain significance (VUS) in mismatch repair (MMR) genes. The presence of all INT^2^GRATE evidence enables the full assessment of a given germline MMR variant. The absence of INT^2^GRATE evidence leads to multiple different scenarios and partial assessment of the variant as described in **Supplementary Tables 1**–**5**. MSI denotes microsatellite instability (MSI).

Four types of well-established clinical evidence were used in the development of INT^2^GRATE decision tree: 1) Presence of a single germline variant in an MMR gene; 2) Qualifying clinical criteria for LS per PREMM5 (13) or Amsterdam II (14); 3) Tumor-derived genetic information, including somatic inactivation of MMR loci, *BRAF* p.V600E status (only for CRC), *MLH1* promoter methylation, and microsatellite instability status; and 4) IHC staining of MMR proteins in tumor cells. Tumor-derived evidence is routinely used in the diagnosis of LS (5, 15). The specific logic used by INT^2^GRATE is detailed in **Supplementary Tables 1**–**5**. Additional information on INT^2^GRATE criteria and the detailed rationale for their inclusion in the decision logic are discussed in the Methods below. A diagrammatic representation of the INT^2^GRATE variant assessment process is shown in **Figure 1**.

### Assignment of INT^2^GRATE categories

INT^2^GRATE decision tree requires the presence of all key parameters for a full assessment of germline VUS. After assessing evidence for a given VUS, INT^2^GRATE renders one of three possible categories accompanied by an explanatory comment. INT^2^GRATE POSITIVE is designated for scenarios in which the evidence supports a likely relevance of the germline variant to LS. INT^2^GRATE NEUTRAL category is rendered when all evidence types in the decision tree are present, but the pattern may be unusual, or rare so that a conservative evaluation of VUS cannot be accomplished. **Supplementary Tables 2**–**5** list details of INT^2^GRATE logics and comments for each gene and scenario. The conservative decision tree logic favors a higher degree of confidence in positive results. INCONCLUSIVE category is rendered when one or more key evidence is absent and INT^2^GRATE excludes the assessment of VUS (**Supplementary Table 1**).

### INT^2^GRATE evidence-based logic and rationale

This section describes the details of the criteria and the rationale behind the inclusion and exclusion of related evidence in the design of the INT^2^GRATE decision tree logic.

## Germline VUS in MMR genes and rationale

Pathogenic variants in an MMR gene or a 3’ deletion of the *EPCAM* gene establish the diagnosis of LS. Therefore, the presence of a VUS in any MMR gene is important information to incorporate into the decision algorithm for the potential role of the germline VUS. A conservative approach was taken in the design of the INT^2^GRATE logic. The application of INT^2^GRATE is contingent upon the presence of only one germline VUS in one MMR gene (**Supplementary Table 1**, INT^2^GRATE CRC Code I-I and EC Code I-I). Also, the presence of 3’ deletion of the *EPCAM* is an exclusion criterion for the assessment of germline VUS by INT^2^GRATE. These conditions exclude the possibility of potentially assessing multiple factors contributing to LS. The presence of possible cryptic rearrangements in MMR genes, not detectable by routine methods, is possible. However, in these cases, the presence of additional complementary evidence from tumor and IHC patterns would help delineate the role of the germline VUS, either by affirming or ruling out its role in LS.

## Clinical criteria and rationale

The presence of clinical criteria is a requirement in the design of INT^2^GRATE (**Supplementary Table 1**, INT^2^GRATE CRC Code I-II and EC Code I-II). Of the criteria currently used in clinical practice, the presence of Amsterdam II (14) or PREMM5 (13) criteria was incorporated into the INT^2^GRATE design. This conservative approach was taken to exclude germline alterations with low penetrance. While VUS alterations with reduced penetrance may well be involved in the development of LS, large-scale data analysis will be required to elucidate their clinical significance. Clinical criteria were also used to evaluate cases in which more than one somatic variant is detected in the same MMR gene. The presence of a strong personal and/or family history with concordant tumor data is highly indicative of LS. Conversely, without such history, sporadic biallelic somatic inactivation is a likely cause of MMR-deficient tumors. Other clinical criteria were not used in the INT^2^GRATE design, and the rationale is discussed below.

***Amsterdam II***

The establishment of testing criteria relied on personal and family history of cancer. Amsterdam I criteria (also called 3-2-1) is the most stringent criteria and states that families should have at least three relatives with colorectal cancer (CRC) in two generations with one diagnosis under the age of 50 and one being a first-degree relative of the other two, and familial adenomatous polyposis excluded (16). Amsterdam II criteria were later established to include other Lynch-associated cancers, including endometrial, small bowel, ureter, or renal pelvis (14). Amsterdam II for Lynch requires at least three relatives should have a Lynch syndrome-associated cancer, one of these three relatives should be a first-degree relative of the other two, at least two successive generations should be affected, at least one relative should be diagnosed before age 50 years, and, for cases of colorectal cancer, familial adenomatous polyposis should be excluded. Because of its stringent criteria, Amsterdam II is used in the design of INT^2^GRATE herein, but only for the assessment of germline VUS in LS-related CRC and endometrial cancer (EC).

*PREMM5*

In the 2000s, as clinical testing became more readily available, data from tested LS cohorts were incorporated into different risk models. These models included MMRPredict, MMRPro, and PREMM (17–19). All three current versions are listed in the National Comprehensive Cancer Network’s Guidelines on Genetics/Familial Risk Assessment for Colorectal Cancer as viable risk calculation models for LS with a suggestion of risk score ≥ 5% as a threshold for testing. Of these, PREMM5, which has since incorporated data on all five LS genes, defines risk scores of ≥ 2.5% as acceptable scores in patients with colorectal cancer/endometrial cancer and possibly unaffected patients, with the caveat that increasing sensitivity may decrease specificity (NCCN v.1.2022). PREMM5 (13) is well-validated and easy to use on a publicly available website. Given the wide acceptance of PREMM5, this model has been incorporated into INT^2^GRATE as part of clinical testing criteria.

***Other criteria not used in INT^2^GRATE design***

Bethesda guidelines were developed to determine which individuals with colorectal cancer should undergo analysis through microsatellite instability testing (20). Revised guidelines included the presence of colorectal cancer under the age of 50, two or more LS-related tumors regardless of age, colorectal cancer diagnosed in patients with less than 60 years of age with MSI-H histology (tumor-infiltrating lymphocytes, Crohn’s like lymphocytic reaction, mucinous or signet-ring differentiation, or medullary growth pattern), colorectal cancer in a patient with ≥1 first-degree relative with LS-related cancer and one diagnosis before age 50, or colorectal cancer in a patient with >2 first- or second-degree relatives with LS-related cancer regardless of age. Several quantitative methods provide risk calculation for the likelihood of having LS. Early versions of these multi-variable models included the Wijnen model and the Amsterdam plus model (21, 22). Both models used the fulfillment of Amsterdam criteria in addition to two to five other characteristics, including the presence of endometrial cancer in the family. Limitations of these models include relatively small sample populations and lack of incorporation of extra-colonic cancers other than endometrial (23). Therefore, these criteria were not used in the design of the INT^2^GRATE.

## Tumor-derived signature data and rationale

Tumor-derived information is a key element in INT^2^GRATE design for the assessment of germline VUS. These criteria, currently used in clinical practice, were used in the design of INT^2^GRATE:

***Somatic BRAF status***

*BRAF* variant status is frequently assessed in colorectal cancer given its prognostic and treatment implications in the MMR proficient (MMRp) colorectal cancer (24–27). As a result, *BRAF* variant testing may be more readily available across a broader range of practice settings than multigene next-generation sequencing (NGS) panels. Similarly, some institutions routinely test for *BRAF* variants, instead of or in addition to *MLH1* promoter methylation, in colorectal cancer with MLH1/PMS2 loss by IHC (28).

Somatic *BRAF* p.V600E variants are exceedingly rare in LS colorectal cancer, but are present in approximately half of sporadic mismatch repair deficient (MMRd) colorectal cancers (29, 30). The presence of *BRAF* p.V600E has been widely used in clinical practice to distinguish LS colorectal cancer and sporadic colorectal cancer (NCCN, 2022) (31). In keeping with clinical practice, colorectal tumors bearing *BRAF* p.V600E are excluded from VUS reassessment with INT^2^GRATE (**Supplementary Table 1**, INT^2^GRATE CRC Code I-III). Furthermore, the significance of *BRAF* alterations other than the p.V600E in LS is less clear. *BRAF* variants are rare in endometrial cancer and are not routinely assessed in the MMRd endometrial cancer (32–42). Therefore, *BRAF* status in endometrial cancer is not a component of INT^2^GRATE (**Supplementary Table 1**).

***Somatic MLH1 methylation status***

Methylation of the *MLH1* promoter is the most common cause of MMRd and microsatellite instability (MSI) in both colorectal cancer and endometrial cancer and is routinely evaluated in tumors that show loss of MLH1 and PMS2 by IHC (41, 43, 44). Tumors with MMRd secondary to *MLH1* promoter methylation are thought to arise sporadically rather than in the setting of LS, with rare exceptions. Whereas BRAF testing will identify approximately half of sporadic MMRd colorectal cancer cases, *MLH1* promoter methylation will definitionally identify sporadic MMRd colorectal cancer. Therefore, negative *MLH1* promoter methylation testing is required to proceed with this VUS reassessment approach (**Supplementary Table 1**, INT^2^GRATE CRC Code I-IV, and EC Code I-III).

***Biallelic inactivation at MMR loci***

Somatic inactivation at MMR loci is the expected mechanism for tumorigenesis in LS (45). If the germline variant in an MMR gene is in fact pathogenic, the remaining functional allele is often preferentially lost through an acquired inactivating single nucleotide or copy number alteration. For this reason, the presence of somatic inactivation of the wild-type allele at MMR loci is required evidence in the application of the INT^2^GRATE (**Supplementary Table 1**, INT^2^GRATE CRC Code I-V and EC Code I-IV). Of note, this criterion may limit the assessment of VUS in many scenarios. For instance, the somatic alteration may be cryptic or undetectable because of assay or technical limitations. This could include somatic epigenetic or structural variants not detected by standard assays. It is also worth noting that it is often unclear if the germline and somatic variants are in cis or trans; however, the somatic and germline variants are presumed to be in trans when the data collectively indicates that this is the most likely situation (e.g., a somatic variant in *PMS2* is identified in a patient with a germline *PMS2* VUS and isolated loss of PMS2 in tumor nuclei). Nevertheless, we have chosen to implement this rule as a required criterion in order to document biallelic inactivation as best as possible with the available findings.

***MMR/MSI tumor signature***

MMR deficiency manifests functionally as MSI, which can be detected by MSI polymerase chain reaction (PCR) or NGS assays. Documentation of MMRd is a requirement for this variant assessment approach (**Supplementary Table 1**, INT^2^GRATE CRC Code I-VI, and EC Code I-V). When possible, this molecular phenotype should be addressed by NGS-based assays, as they allow for the simultaneous estimation of tumor mutational burden, the detection of mutations in the MMR and *BRAF* genes, and the assessment of copy-number status. Whereas PCR-based assays usually examine long mononucleotide and/or dinucleotide tracts in non-coding regions, NGS-based assays can additionally assess microsatellites in coding regions targeted by the particular assay. At our institution (Brigham and Women’s Hospital, BWH), we assess for single nucleotide indels in mononucleotide tracts with a length of at least 4 nucleotides (46). Lastly, as is the case in our institution, NGS-based assays can be performed on tumor samples without paired normal tissue.

## MMR IHC pattern and rationale

An essential component of INT^2^GRATE is the presence of supportive IHC data. IHC for MMR proteins is routinely used to screen cases of CRC and EC for MMRd. Loss of IHC expression of at least one MMR protein is observed in most MMRd cases and can indicate biallelic inactivation of one of the MMR proteins in certain scenarios. Therefore, evaluation of the four main MMR proteins by IHC and loss of IHC expression of at least one MMR protein are both required for this approach (**Supplementary Table 1**, INT^2^GRATE CRC Code I-VII and EC Code I-VI). These requirements limit the utility of INT^2^GRATE in assessing VUS that may occur in rare cases exhibiting MSI-H but intact MMR IHC (47, 48) and in cases in which only PMS2 and MSH6 IHC was performed. This conservative approach will allow for a higher degree of confidence in the results of our integrated VUS reassessment. Furthermore, the IHC pattern can support the presence of a defect in a particular MMR gene.

## Development of INT^2^GRATE Web UI & data sharing features

The INT^2^GRATE UI (available at https://INT2GRATE.bwh.harvard.edu/) was designed to enable an intuitive capturing of user selections, to assess VUS according to logics displayed in **Supplementary Tables 1**–**5**, and to facilitate data and knowledge sharing by providing users an option for submitting their entry and INT^2^GRATE results to ClinVar database. Possible scenarios were implemented in Javascript conditional logic statements that return the result based on the combination of answers selected by the user.

***Design of the INT^2^GRATE digital UI***

**Figure 2:**
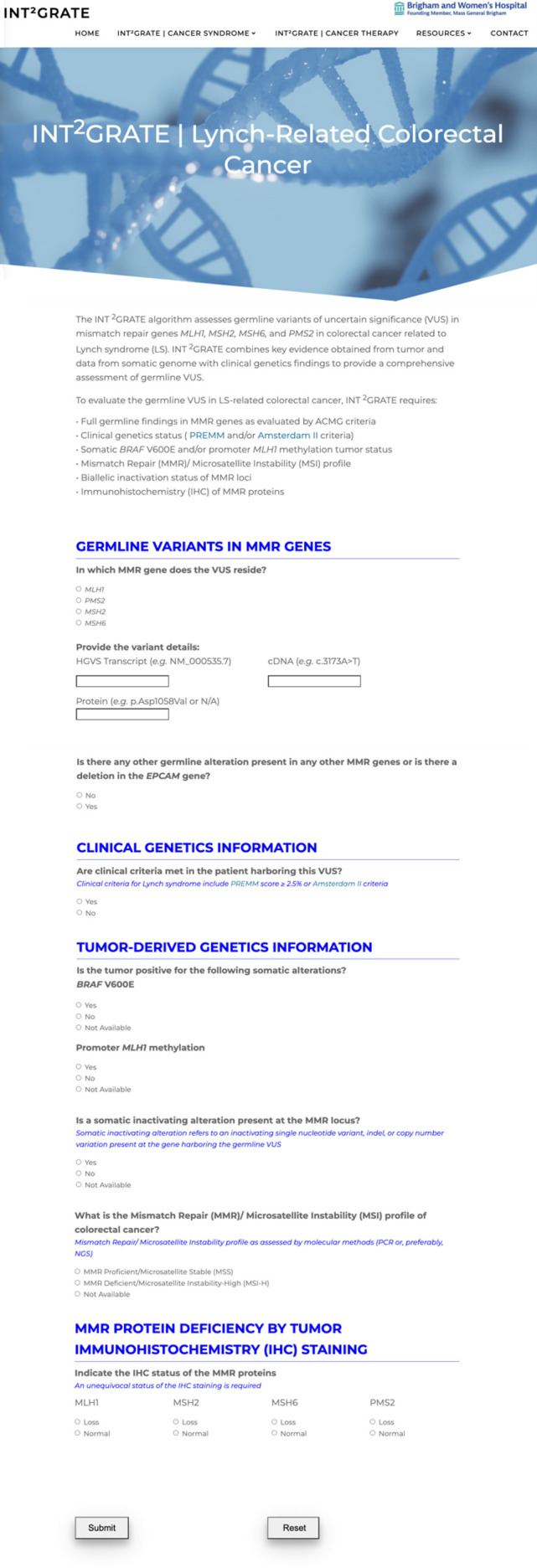
The INT^2^GRATE Web-Based User Interface (UI) for Lynch-Related Colorectal Cancer. INT^2^GRATE UI enables an intuitive collation of key evidence for the assessment of germline VUS. Upon completion of entries and pressing Submit, the assessment is performed in the backend according to the INT^2^GRATE logic, and an INT^2^GRATE category and explanation are displayed. The UI provides users with an option for downloading their entry and the INT^2^GRATE results and sharing them in the public ClinVar database.

**Figure 3:**
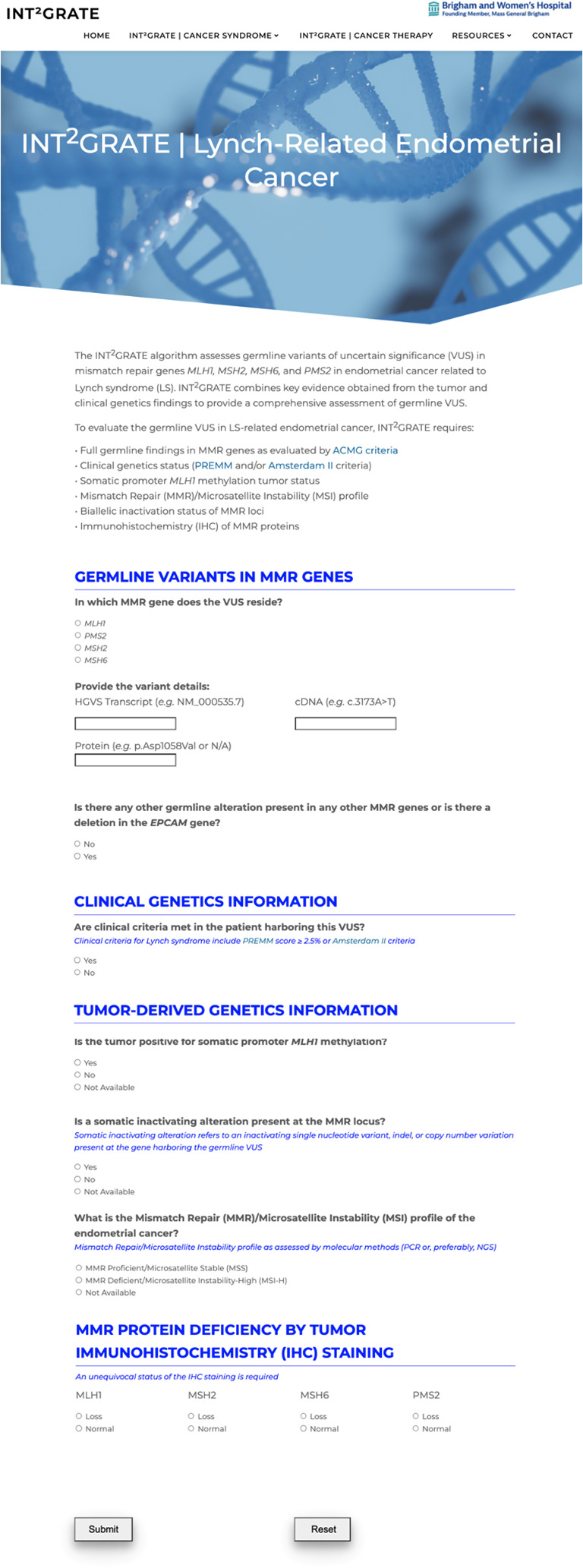
The INT^2^GRATE Web-Based User Interface (UI) for Lynch-Related Endometrial Cancer. INT^2^GRATE UI enables an intuitive collation of key evidence for the assessment of germline VUS. Upon completion of entries and pressing Submit, the assessment is performed in the backend according to the INT^2^GRATE logic, and an INT^2^GRATE category and explanation are displayed. The UI provides users with an option for downloading their entry and the INT^2^GRATE results and sharing them in the public ClinVar database.

The digital interface for INT^2^GRATE is a web-based application that allows the user to assess germline VUS using a collection of relevant somatic and clinical evidence in selected cancer types. From the main page for the website (https://INT2GRATE.bwh.harvard.edu/), the user can select Lynch Syndrome-Related Colorectal Cancer or Lynch Syndrome-Related Endometrial Cancer to access their respective interfaces hosted in separate webpages of the site. Each page presents the algorithm-dependent questions (11 for Colorectal and 10 for Endometrial) via radio button selection to provide single response input. The front-end UI was designed in an HTML form to allow the user to select their entry and receive the appropriate assessment as dictated by the logical combination (**Figures 2**, **3**). A quality control measure was implemented so that the INT^2^GRATE UI does not process possible entries outside of **Supplementary Tables 1**–**5** of the current algorithm. The algorithm runs on Javascript in the client-side (i.e., user’s) browser and does not interface with a server to perform this logic. User information and entries are not sent to or stored in servers. The website is accessible through any web browser.

***Display of the INT^2^GRATE results***

After the completion of questions and pressing submit, responses are processed as Javascript variables according to the decision tree logic (**Supplementary Tables 1**–**5**) to determine the INT^2^GRATE categories and relevant comments. The response will conditionally return only one of three possible INT^2^GRATE categories depending on the combination of responses: a) INT^2^GRATE POSITIVE, b) INT^2^GRATE NEUTRAL, and c) INCONCLUSIVE (categories are described in the Assignment of INT^2^GRATE Categories section). The details of combinations that produce these three categories are outlined in **Supplementary Tables 1**–**5**. If the logical combination is not provided within the current algorithm, a response populates at the bottom of the page that indicates the logical combination falls outside of the current parameters of the algorithm.

***Facilitating data sharing***

After evidence collection and VUS assessment are performed and an INT^2^GRATE category is returned from the algorithm, the user has the option to download a synopsis of the questions and their responses for INT^2^GRATE POSITIVE and INT^2^GRATE NEUTRAL cases. The downloaded file is accessible in a spreadsheet. This file and the information captured are stored locally on the user’s computer; no information is collected by external servers or shared with external sources as a result of algorithm use.

To facilitate submission and data sharing in the ClinVar database, the submission file features several fields prompted by ClinVar for variant submission as cells in the spreadsheet, which can be directly copied into ClinVar’s submission form by the user. This synopsis will capture fields required by ClinVar including the Assertion Method, Assertion Method Citation, Citation URL, and Comment on Clinical Significance, as well as the date of download from INT^2^GRATE and the version of the algorithm used in the variant’s assessment on that date. The user can include additional information to the evidence summary (e.g., ACMG criteria) before submitting it to ClinVar.

***Tagging downloaded files by variants***

All fields on the INT^2^GRATE webpage are required to be filled out by the user for INT^2^GRATE to assess germline VUS. Additionally, the user is required to enter the specific variant being assessed in a field prompting the cDNA. The variant, gene, and cDNA will be automatically tagged to the file to be downloaded on the user’s computer to help identify different variants assessed by the user. An optional field for the protein change is provided and will be included in the file download if provided by the user.

### Assessment of INT^2^GRATE Performance

#### Patients, tumors, and genetic characteristics

Two clinic-based cohorts were used to evaluate the performance of INT^2^GRATE. The first cohort consisted of 5018 patients with MMR single nucleotide variants (SNV) and/or copy number variants (CNV) detected by the somatic OncoPanel next-generation sequencing (NGS) assay from the Center for Advanced Molecular Diagnostic at Brigham and Women’s Hospital between 2018 and 2022. For each case, tumor MSI status, tumor mutational burden, tumor type, and all reported variants in OncoPanel’s 447 genes were collected. Germline testing was performed for 348 of these individuals by Dana Farber Cancer Institute (DFCI) for clinical genetics evaluation. Thirteen of these patients were diagnosed with LS-related CRC or EC and carried a pathogenic variant in an MMR gene.

The second cohort comprised of patients evaluated at DFCI for LS between 2017 and 2022, tested by TumorNext Lynch (Ambry Genetics, CA, USA) for both germline and tumor profiles, and diagnosed with LS-related CRC or EC. Thirty-nine patients met these criteria with a pathogenic germline MMR variant. Germline and somatic genetic analysis and IHC staining were performed as described previously (9, 10, 49) and as outlined in the **Supplementary Laboratory Methods**.

### Development of comprehensive somatic and germline databases

A Comprehensive Somatic Variant Database (CSVD) was generated using the R programming language to include SNV, CNV, and structural variant data for each case, along with additional tumor-related information including tumor type and MSI and BRAF status. A second database, the Integrated Somatic, and Germline Variant Database, was generated in “R” by merging the germline variant data obtained from DFCI with the CSVD.

## Results

### INT^2^GRATE assessment of germline VUS in *MLH1*

*MLH1* germline VUS is potentially relevant to LS when each of the following criteria are met: The *MLH1* VUS is the only germline alteration in MMR genes; no other germline alteration related to LS is present; clinical criteria (i.e. PREMM score or Amsterdam II) are met; somatic *MLH1* promoter methylation and/or *BRAF* p.V600E are absent; somatic inactivation of the second *MLH1* allele is documented; microsatellite instability is identified by PCR or NGS; and a compatible IHC staining pattern is present (**Table 1**, INT^2^GRATE POSITIVE CRC Codes II-I to II-IV and EC Codes II-I to II-II). Typically, these cases would show loss of MLH1 and PMS2 in tumor nuclei, as biallelic inactivation of *MLH1* would result in loss of MLH1 and its dimerization partner PMS2. Isolated loss of PMS2 in tumor nuclei would also be compatible in these cases, as *MLH1* alterations can also lead to retained expression of nonfunctional MLH1 with loss of PMS2 (47, 50). All INT^2^GRATE POSITIVE scenarios and detailed comments are outlined in **Supplementary Table 2**.

**Table 1:** Performance assessment of INT^2^GRATE in LS-related colorectal cancer.

Subjects	PREMM score	Germline alteration	MLH1 hypermethylation	MSI	IHC	INT^2^GRATE Code ID
		Inactivating somatic allele				
30	5.90%	*MSH2*:EX12_15del	Neg	MSI-High	loss of MSH2 and MSH6	IV-I
		*MSH2*:c.1697dupA (p.N566Kfs*2)				
31	6.70%	*MSH2*:c.226C>T (p.Q76*)	Neg	MSI-High	loss of MSH2 and MSH6	IV-I
		*MSH2*:c.2334C>A (p.C778*)				
32	4.20%	*MSH6*:c.3436C>T (p.Q1146*)	Neg	MSI-High	loss of MSH6	V-I
		*MSH6*:c.3238_3239delCT (p.L1080Vfs*12)				
33	3.00%	*MSH6*:c.3416G>T (p.G1139V)	Neg	MSI-High	loss of MSH6	V-I
		*MSH6*:c.3261delC (p.F1088Sfs*2)				
34	2.50%	*MSH6*:c.3984_3987dupGTCA (p.L1330Vfs*12)	Neg	MSI-High	loss of MSH6	V-I
		*MSH6*:c.2933delA (p.Q978Rfs*19)				
35	4.00%	*PMS2*:c.736_741delCCCCCTins11 (p.P246Cfs*3)	Neg	MSI-High	loss of PMS2	III-I
		*PMS2* c.1376_1405del30 (p.S459*)				
36	2.90%	*PMS2*:c.2192_2196del (p.L731Cfs*3)	Neg	MSI-High	loss of PMS2	III-I
		*PMS2* one copy loss				
37	7.00%	*MSH2*:c.309T>A (p.Y103*)	Neg	MSI-High	loss of MSH2 and equivocal MSH6**	IHC pattern not included in INT^2^GRATE assessment for VUS
		*MSH2* single copy loss				
38	2.00%	*PMS2*:c.2117delA (p.K706Sfs*19)	Positive	MSI-High	loss of MLH1 and PMS2	I-II, I-III, or I-IV
		None Detected				
39	5.40%	*PMS2*:c.767delG ( p.G256Vfs*2)	Neg	MSI-High	loss of PMS2 and MSH6	I-IV
		None Detected				
40	2.4%	*MSH6*:c.3261dupC (p.F1088Lfs*5)	Neg	MSI-High	loss of MSH6	I-II or I-IV
		None Detected				
41	5.60%	*MSH6*:c.3261delC (p.F1088Sfs*2)	Neg	MSI-High	loss of MSH6	I-IV
		None Detected				
42	6.20%	*MSH6*:c.3939_3957dup19 (p.A1320Sfs*5)	Neg	MSI-High	loss of MSH6	I-IV
		None Detected				
43	4.70%	*PMS2*:c.137G>T (p.S46I)	Neg	MSI-S	loss of PMS2	I-IV or I-V
		None Detected				
44	2.70%	*MSH6*:c.10C>T (p.Q4*)	N/A	MSI-High	Loss of MSH6	I-IV
		None Detected				
45	3.30%	*PMS2*:c.861_864delACAG (p.R287Sfs*19)	Neg	MSI-High	loss of PMS2	I-IV
		*PMS2*:c.404T>C (p.L135P)				
46	3.10%	*PMS2*:c.137G>T (p.S46I)	Neg	MSI-High	loss of PMS2	I-IV
		*PMS2*:c.163+5G>A				
47	14.80%	*MSH2*:c.2152C>T (p.Q718*)	Neg	MSI-High	loss of MSH2 and MSH6	I-IV
		*MSH2*:c.1241_1255del15 (p.L414_I418del)				
48	2.00%	*PMS2*:EX9_10del	Neg	MSI-High	loss of PMS2	I-II
		*PMS2* single copy loss				
49	2.4%	*MSH6*:c.3268_3274delGAGCTTA (p.E1090Kfs*23)	N/A	MSI-High	Loss of MSH6	I-II
		*MSH6*:c.3731T>G (p.L1244*)				
50	7.00%	*MSH6*:c.10C>T (p.Q4*)	Neg	None	loss of MSH6	I-V
		*MSH6*:c.2386G>T (p.E796*)				
51	5.70%	*MSH2*:c.1906G>C (p.A636P)	Neg	None	loss of MSH2 and MSH6	I-V
		*MSH2*:c.1861C>T (p.R621*)				
52	5.10%	*MSH6*:c.742C>T (p.R248*)	Neg	MSI-S	loss of MSH6	I-V
		*MSH6*:c.3261delC (p.F1088Sfs*2)				

IHC, Immunohistochemistry; LOH, Loss of heterozygosity; MMR, Mismatch repair; MMR-D, Mismatch repair-deficient; MMR-P, Mismatch repair-proficient; MSI, Microsatellite instability; MSI-H, Microsatellite instable-high.

**Partial or equivocal IHC pattern not incorporated in INT^2^GRATE assessment.

Any unusual pattern of evidence or deviation from the above stringent criteria would render the designation of INT^2^GRATE NEUTRAL (i.e., the significance of VUS remains uncertain in relation to LS). The details of these scenarios and associated comments are outlined in **Supplementary Table 2**. For CRC, the significance of VUS remains uncertain if *MLH1* promoter methylation and/or *BRAF* testing are positive, if results for both tests are unavailable, or if IHC shows an unusual staining pattern (i.e., isolated loss of MLH1) (**Supplementary Table 2**, INT^2^GRATE CRC Codes II-V to II-VIII). While *MLH1* promoter methylation testing is preferred (see sections on *BRAF* and *MLH1* promoter methylation status in **Supplementary Methods**), a negative *BRAF* result in the absence of *MLH1* promoter methylation testing is acceptable for VUS assessment granted all other conditions are met. Confirmatory *MLH1* promoter methylation testing should be considered in this scenario. For EC, the significance of VUS remains uncertain if *MLH1* promoter methylation is positive, if results for this test are unavailable, or if IHC shows an unusual staining pattern (**Supplementary Table 2**, INT^2^GRATE EC Codes II-1II to II-V). Isolated loss of MLH1 is unusual; this IHC pattern would warrant a review of the IHC findings and potentially even repeat IHC.

### INT^2^GRATE assessment of germline VUS in *PMS2*

*PMS2* germline VUS is potentially relevant to LS when each of the following criteria are met: The *PMS2* VUS is the only germline alteration in MMR genes; no other germline alteration related to LS is present; clinical criteria (i.e. PREMM score or Amsterdam II) are met; somatic *MLH1* promoter methylation and/or *BRAF* p.V600E are absent; somatic inactivation of the second *PMS2* allele is documented; microsatellite instability is identified by PCR or NGS; and loss of only PMS2 in tumor nuclei by IHC is present (**Supplementary Table 3**, INT^2^GRATE POSITIVE CRC Codes III-I to III-III, and EC Code III-I). In these cases, isolated loss of PMS2 by IHC would support biallelic inactivation of the *PMS2* gene.

The significance of VUS remains uncertain if both MLH1 and PMS2 are lost by IHC, regardless of results for *MLH1* promoter methylation and/or *BRAF* testing, as this pattern would suggest that the *MLH1* gene is altered. (INT^2^GRATE NEUTRAL CRC Codes III-IV to III-VII, and EC Codes III-II to III-III).

### INT^2^GRATE assessment of germline VUS in *MSH2*

*MSH2* germline VUS is potentially relevant to LS when each of the following criteria is met: The *MSH2* VUS is the only germline alteration in MMR genes; no other germline alteration related to LS is present; clinical criteria (i.e., PREMM score or Amsterdam II) are met; somatic *MLH1* promoter methylation and/or *BRAF* p.V600E are absent; somatic inactivation of the second *MSH2* allele is documented; microsatellite instability is identified by PCR or NGS; and IHC shows a compatible staining pattern (**Supplementary Table 4**, INT^2^GRATE POSITIVE CRC or EC Codes IV-I to IV-II). Typically, these cases would show loss of MSH2 and MSH6 as a result of biallelic inactivation of *MSH2* and degradation of its dimerization partner. Isolated loss of MSH6 in tumor nuclei would also be compatible, as certain *MSH2* alterations lead to retained MSH2 expression with loss of MSH6 (47).

The significance of VUS remains uncertain if there is an unusual IHC pattern showing an isolated loss of MSH2 (**Supplementary Table 4**, INT^2^GRATE NEUTRAL Code IV-III). In such cases, a review of the IHC findings is recommended, as MSH6 may not be uniformly lost with some IHC assays. If all MMR proteins are lost by IHC, a more detailed examination of the IHC results should be performed, as the loss of MLH1 and PMS2 could be subclonal (**Supplementary Table 4**, INT^2^GRATE NEUTRAL Code IV-III); the significance of VUS in these scenarios remains uncertain.

### INT^2^GRATE assessment of germline VUS in *MSH6*

*MSH6* germline VUS is potentially relevant to LS when each of the following criteria is met: The *MSH6* VUS is the only germline alteration in MMR genes; no other germline alteration related to LS is present; clinical criteria (i.e., PREMM score or Amsterdam II) are met; somatic *MLH1* promoter methylation and/or *BRAF* p.V600E are absent; somatic inactivation of the second *MSH6* allele is documented; microsatellite instability is identified by PCR or NGS; and only MSH6 is lost in tumor nuclei by IHC (**Supplementary Table 5**, INT^2^GRATE POSITIVE CRC or EC Code V-I). Absent MSH6 on IHC would support biallelic inactivation of the *MSH6* gene.

Deviations from these criteria render the designation of INT^2^GRATE NEUTRAL. Loss of both MSH2 and MSH6 in tumor nuclei by IHC would suggest a likely alteration in *MSH2* (**Supplementary Table 5**, INT^2^GRATE NEUTRAL Code V-II). Loss of MSH6, MLH1, and PMS2 in tumor nuclei by IHC would suggest a secondary loss of MSH6 (51) (**Supplementary Table 5**, INT^2^GRATE NEUTRAL Code V-III). The significance of VUS in these scenarios remains uncertain.

### Efficacy of INT^2^GRATE performance

The performance of INT^2^GRATE was assessed by a qualitative retrospective analysis of data obtained from 5057 patients from two clinic-based cohorts (n_1 =_ 5018, and n_2 =_ 39), including a total of 52 patients with LS-related CRC or EC and known pathogenic MMR variants that served as positive controls.

Among the 52 LS patients with known pathogenic MMR variants, 23 had all evidence or parameters for the evaluation by INT^2^GRATE. Each of these 23 variants was categorized as INT^2^GRATE POSITIVE (**Tables 1**, **2**; **Supplementary Tables 1**, **2**). In the positive control cohort, the personal and family history of these cases was strongly supportive of Lynch syndrome (**Table 2**). Demographics and Clinical Characteristics of Lynch Syndrome Subjects in Positive Control Cohorts are presented in **Table 3**.

**Table 2:** Performance assessment of INT^2^GRATE in LS-related endometrial cancer.

Subjects	PREMM score	Germline alteration	BRAF V600E, MLH1 hypermethylation	MSI	IHC	INT^2^GRATE Code ID
		Inactivating somatic allele				
1	5.70%	*MLH1*:c.116+2T>G	Neg, Unmethylated *MLH1* promoter	MSI-High	Loss of MLH1 and PMS2	II-III
		*MLH1* single-copy loss				
2	36.40%	*MLH1*:c.1783_1784delAG (p.S595Wfs*14)	Neg, NA	MSI-High	Loss of MLH1 and PMS2	II-I
		*MLH1*:c.790+5G>A				
3	7.2%	*MSH2*:c.704_705del (p.K235Rfs*20 )	Neg, NA	MSI-High	Loss of MSH2 and MSH6	IV-I
		*MSH2*:c.2446C>T (p.Q816*)				
4	5.00%	*MSH2*:c.340G>T (p.E114*)	Neg, NA	MSI-High	Loss of MSH2 and MSH6	IV-I
		*MSH2*:c.657delA (p.G220Efs*4)				
5	22.3%	*MSH2*:c.2041C>T (p.Q681*)	Neg, NA	MSI-High	Loss of MSH2 and MSH6	IV-I
		*MSH2*:c.2260_2269del (p.S755Mfs*5)				
6	7.40%	*MSH2*:c.2459-12A>G	Neg, NA	MSI-High	Loss of MSH2 and MSH6	IV-I
		*MSH2*:c.2090G>T (p.C697F)				
7	3.50%	*MSH2*:c.1906G>C (p.A636P)	Neg, NA	MSI-High	Loss of MSH2 and MSH6	IV-I
		*MSH2*:c.1861C>T (p.R621*)				
8	3.90%	*MSH2*:c.942+3A>T	Neg, NA	MSI-High	Loss of MSH2 and MSH6	IV-I
		*MSH2*:c.2131C>T (p.R711*), *MSH2* single-copy loss				
9	32.10%	*MSH2*:c.2038C>T (p.R680*)	Neg, NA	MSI-High	Loss of MSH2 and MSH6	IV-I
		*MSH2*:c.2362dupA (p.T788Nfs*11)				
10	4.30%	*MSH2* EXON7 copy gain (5 copies)	Neg, NA	MSI-High	Loss of MSH2 and MSH6	IV-I
		*MSH2*:c.1216C>T (p.R406*)				
11	20.1%	*MSH2*:c.2131C>T (p.R711*)	Neg, NA	MSI-High	Loss of MSH2 and MSH6	IV-I
		*MSH2* single-copy loss				
12	[9.7%]	*MSH2*:c.2314delA (p.T772Qfs*40)	Neg, NA	MSI-High	Loss of MSH2 and MSH6	IV-I
		*MSH2* single-copy loss				
13	2.50%	MSH6:c.10C>T (p.Q4*)	Neg, NA	MSI-High	Loss of MSH6	V-I
		MSH6:c.1810G>T ( p.E604*), MSH6:c.3261delC (p.F1088Sfs*2)				
14	6.40%	MSH6:c.2230dupG (p.E744Gfs*12)	Neg, NA	MSI-High	Loss of MSH6	V-I
		MSH6:c.3261dupC (p.F1088Lfs*5)				
15	11.60%	*PMS2*:c.137G>T (p.S46I)	Neg, NA	MSI-High	Loss of PMS2	III-III
		*PMS2*:c.163+2T>C, *PMS2*:c.2444C>T (p.S815L)				
16	14.40%	*PMS2*:c.2192_2196delTAACT (p.L731Cfs*3)	Neg, NA	MSI-High	Loss of PMS2	III-III
		*PMS2*:c.2444C>T (p.S815L)				
17	2.70%	*PMS2* 5'UTR_EX15del	Neg, NA	MSI-High	Loss of PMS2 and partial loss MSH6**	IHC pattern not included in INT^2^GRATE assessment for VUS
		*PMS2*:c.1663C>T (p.Q555*)				
18	3.20%	*PMS2*:c.861_864delACAG (p.R287Sfs*19)	Neg, NA	MSI-High	Loss of PMS2	I-V
		None Detected				
19	>50%	*MSH2*:c.1226_1227delAG (p.Q409Rfs*7)	Neg, NA	MSI-High	Loss of MSH2 and MSH6	I-V
		None Detected				
20	20.3%	*MLH1*:c.2142G>A (p.W714*)	Neg, NA	MSI-High	Loss of MLH1 and PMS2	I-V
		None Detected				
21	11.80%	*PMS2*:c.736_741delCCCCCTins11 (p.P246Cfs*3)	Neg, NA	MSI-High	Loss of PMS2	I-V
22	45.10%	*MLH1*:c.677G>A (p.R226Q)	Neg, NA	MSI-High	Loss of MLH1, MSH6, and PMS2	I-V
		None Detected				
23	0.60%	*PMS2*: c.861_864delACAG (p.R287Sfs*19)	Neg, NA	MSI-High	Loss of PMS2	I-II
		*PMS2*:5'UTR_EX5del				
24	1.60%	*PMS2*:c.2445+1G>T	Neg, NA	MSI-High	Loss of PMS2	I-II
		*PMS2*:c.2404C>T (p.R802*)				
25	1.30%	MSH6:c.3261delC (p.F1088Sfs*2)	Neg, MLH1 promoter hyper methylation	MSI-High	Loss of MSH6	I-II, I-IV for *MLH1* Promoter h
		MSH6:c.538delG(p.D180Mfs*4)				
26	1.20%	*PMS2* EXON10del	Neg, NA	MSI-High	Loss of PMS2	I-II
		*PMS2*:c.2174+1G>A				
27	27.50%	*MLH1*:c.292G>C (p.G98R)	Neg, NA	MSI-High	Normal IHC	I-VII
		*MLH1*:c.3G>C (p.M1?)				
28	38.30%	*MLH1*:c.301G>C (p.G101R)	Neg, NA	MSI-High	Normal IHC	I-VII
		*MLH1*:c.199G>A (p.G67R)				
29	14.60%	MSH6:c.10C>T (p.Q4*)	Neg, NA	MSI-High	Normal IHC	I-VII
		MSH6:c.3622_3625delTCTC (p.S1208Wfs*7)				

IHC, Immunohistochemistry; LOH, Loss of heterozygosity; MMR, Mismatch repair; MMR-D, Mismatch repair-deficient; MMR-P, Mismatch repair-proficient; MSI, Microsatellite instability; MSI-H, Microsatellite instable-high.

[]Adopted.

**Partial or equivocal IHC pattern not incorporated in INT^2^GRATE assessment.

**Table 3:** Demographics and clinical characteristics of subjects in positive control cohorts.

Subjects	Age at dx (current age)	Sex	Self-Reported Ancestry	Colorectal/Endometrial Cancer	Related syndromic tumor in subject and/or family
**Subject 1**	46 (55)	F	Irish, Armenian	Colorectal	Mother- Endometrial cancer age 40; Maternal uncle- Colon cancer age 45
**Subject 2**	35 (39)	F	German, Welsh, English, Syrian	Colorectal	Mother- Colon cancer age 59; Maternal uncle- Colon cancer age 42; Maternal male cousin-Colon cancer age 33; Maternal grandmother- Endometrial cancer age 42 and Colon cancer age 62
**Subject 3**	40 & 55 (56)	F	Irish, Italian, Lithuanian	Endometrial and Colorectal	None
**Subject 4**	54 (60)	M	Polish, Slovakian	Colorectal	Two brothers- Pancreatic cancer ages 50 and 52; Paternal grandfather- Small intestinal cancer age 37; Paternal male first cousin- Small intestinal cancer age 46
**Subject 5**	51 (56)	M	Chinese	Colorectal	Brother- Colon cancer age 52; Mother- Colon cancer age 64; Maternal uncle- Stomach cancer age 63; Maternal aunt- Colon cancer and Stomach cancer both at age 64
**Subject 6**	33 (34)	F	Emirian	Colorectal	Maternal half-uncle- Colon cancer age 53
**Subject 7**	50 (52)	M	Lebanese, Scottish, Ukrainian, Ashkenazi Jewish	Colorectal	Maternal male uncle- Colon cancer age 72
**Subject 8**	55 & 61 (67)	M	Native American, German, Lithuanian	Colorectal	Subject- Ureter cancer age 55&65; Mother- Pancreatic cancer age 53; Sister- Pancreatic cancer age 51
**Subject 9**	32 (36)	M	Irish, Japanese, African American	Colorectal	Mother- Endometrial cancer age 45
**Subject 10**	62 (64)	M	Italian, Irish, French, German	Colorectal	Sister- Ovarian cancer age 44; Father- Stomach cancer age 65
**Subject 11**	43 (48)	M	Unknown (adopted)	Colorectal	N/A
**Subject 12**	29 (37)	F	Indian	Colorectal	N/A
**Subject 13**	51 (57)	F	French-Canadian	Colorectal	N/A
**Subject 14**	46 (51)	M	French, German	Colorectal	None
**Subject 15**	41 (42)	M	Irish, Eastern European, Ashkenazi Jewish	Colorectal	Maternal aunt- Endometrial cancer age 46
**Subject 16**	48 & 64 (69)	F	English, Scottish, Welsh, Austrian, Polish	Endometrial and Colorectal	Brother- Colon cancer age 45; Mother- Bladder cancer age 68
**Subject 30**	40 (52)	F	Portuguese	Endometrial	Paternal aunt- Colon cancer age 58; Two Paternal aunts- Colon cancer unknown ages; Paternal uncle- Colon cancer age 62
**Subject 31**	37 (40)	F	Kuwaiti	Endometrial	Mother- Endometrial cancer age 47
**Subject 32**	71 (76)	F	Irish, English, Scottish, Greek	Endometrial	Subject- Sebaceous adenoma age 69
**Subject 33**	56 (60)	F	Greek	Endometrial	Paternal uncle- GI cancer unknown type; Two paternal male cousins- Colon cancer age 60; Paternal female cousin- Glioblastoma age 46; Paternal male cousin- Glioblastoma age 62; Paternal male cousin- Small intestinal cancer age 58
**Subject 34**	66 (69)	F	Ashkenazi Jewish	Endometrial	Mother- Colon cancer age 82; Maternal uncle- Stomach cancer age 79; Maternal female cousin- Ovarian cancer age 57
**Subject 35**	55 (60)	F	Irish, English, Scottish, African American	Endometrial	Mother- Pancreatic cancer age 53; Maternal uncle- Pancreatic cancer unknown age; Maternal aunt- Pancreatic cancer age unknown age
**Subject 36**	51 (56)	F	Ukrainian	Endometrial	None
**Subject 37**	39 (42)	F	Portuguese, Spanish	Endometrial	Mother- Endometrial cancer age 52
**Subject 38**	75 (80)	F	French	Endometrial	Mother- Colon cancer age 70
**Subject 39**	67 (72)	F	Latvian, German, Russian, Ashkenazi Jewish	Endometrial	Sister- Endometrial cancer age 60; Maternal first cousin- Ovarian cancer age 58; Maternal first cousin once removed- Pancreatic cancer age 45
**Subject 40**	65 (70)	F	Russian, Belgian, Ashkenazi Jewish, Hungarian, Irish, Spanish	Endometrial	None
**Subject 41**	58 (63)	F	Russian, Polish, Lithuanian, Ashkenazi Jewish, English, Irish	Endometrial	Sister- Endometrial cancer age 42; Mother- Kidney cancer age 70 and Glioblastoma age 81; Maternal first cousin- Brain tumor age 27
**Subject 42**	65 (68)	F	English	Endometrial	Father- Colon cancer age 69; Paternal aunt- Endometrial cancer age 85
**Subject 43**	63 (65)	F	Polish, Russian, Irish, German, English	Endometrial	Maternal aunt- Endometrial cancer age 76; Paternal grandmother- Endometrial cancer age 60; Paternal uncle- Brain tumor age 63
**Subject 44**	57 (61)	F	French, Italian	Endometrial	Maternal great grandmother- Colon cancer unknown age
**Subject 45**	50 (56)	F	Irish, Italian, Polish	Endometrial	Paternal grandmother- Brain tumor age 58 and Liver cancer age 85; Paternal great uncle- Pancreatic cancer age 78; Paternal great uncle- Liver cancer age 78
**Subject 46**	47 (52)	F	Italian, French-Canadian	Endometrial	Paternal grandfather- Gastrointestinal cancer unknown age
**Subject 47**	42 (48)	F	Brazilian	Endometrial	Mother- Endometrial cancer age 42; Nephew- Colon cancer unknown age; Maternal aunt- Endometrial cancer age 62; Maternal first cousin- Endometrial cancer age 55 and Stomach cancer age 55 and Brain tumor age 55; Maternal grandmother- Endometrial cancer age 65; Paternal grandmother- Brain tumor age 69
**Subject 48**	74 (77)	F	French-Canadian, Irish	Endometrial	Maternal first cousin- Kidney cancer age 68
**Subject 49**	45 (49)	F	Polish, German, French, Native American	Endometrial	Paternal grandmother- Colon cancer age 65
**Subject 50**	66 (68)	F	French-Canadian	Endometrial	Father- Colon cancer age 47 and Stomach cancer age 95; Paternal uncle- Stomach cancer age 89; Paternal first cousin- Stomach cancer age 74
**Subject 51**	52 (56)	F	Ashkenazi Jewish	Endometrial	Brother- Kidney cancer age 44; Father-Colon cancer age 60 and Small Intestinal cancer age 73
**Subject 52**	57 (73)	F	Irish, Unknown	Endometrial	Mother- Endometrial cancer age 80

The remaining 29 of 52 positive controls with known pathogenic MMR variants did not have all evidence in the INT^2^GRATE decision tree (**Tables 1**, **2**; **Supplementary Tables 1**, **2**). These included 16 variants in the CRC cancer group and 13 in the EC group. The absence of biallelic inactivation was the most frequent observation (n=15), followed by a low PREMM5 score (<2.5%) (n=8), normal, partial, or equivocal IHC (n=4), and unavailable or stable status of the MSI test (n=4) (**Tables 1**, **2**; **Supplementary Tables 1**, **2**). Rare pattern or absence of key evidence was flagged by the INT^2^GRATE assessment by assigning separate ID codes (**Tables 1**, **2**). This highlights the conservative approach in designing INT^2^GRATE for the assessment of VUS in favor of a higher degree of confidence in the results.

In the remaining OncoPanel cohort, INT^2^GRATE logic effectively identified 5005 cases with the absence of ≥ 1 criteria, rendering a classification of INCONCLUSIVE. Overall, 3735 (74%) were MMR proficient/microsatellite stable (MMR-P/MSS) cases. A total of 1339 cases had at least one SNV in the MMR gene, of those 72% (970/1339) showed the absence of biallelic alterations at the MMR locus, and 2% (27/1339) were positive for *BRAF* p.V600E.

### Collation and sharing of INT^2^GRATE evidence

Data sharing is an essential goal of the INT^2^GRATE program. INT^2^GRATE’s user-friendly interface enabled the collection and download of all decision tree evidence in the form of questions and answers by the user. A list of evidence, the associated comment for each scenario (as described in **Supplementary Tables 1**-**5**), and a summary of responses are automatically generated as a text file for each case (**Supplementary Table 6**). This downloadable text file facilitates data sharing or submission of the variant’s evidence summary to ClinVar.

## Discussion

The standard germline sequence variant classification system (6) yields a high number of VUS, the largest type of variants reported in clinical practice. Collectively, there are 10,488 germline VUS in *MLH1, MSH2*, *MSH6*, and *PMS2* in the ClinVar database to date (as of November 2023). The prevalence of VUS is disproportionately higher in underrepresented populations (52–54), partially due to information disparity in genomics, which in turn propagates health inequity in genomics. The economic, clinical, psychosocial, and emotional burden associated with VUS is well documented (55–60). While many tools and strategies aim to assess the functionality or pathogenicity of variants (61–69), none exists that systematically integrates tumor information in the assessment of germline VUS. The absence of joint databases of germline and somatic variant data further limits the utility of integrated information in cancer genetics.

INT^2^GRATE leverages information from the two genomes that exist in patients with cancer, germline and tumor, to inform the potential role of constitutional VUS in MMR genes. This integrated measure is an objective indication of the status of tumor progression at the molecular and cellular level and is arguably free of confounding genetic host factors and factors influenced by population genetic structure. While the majority of individuals in our study identified as having mostly European backgrounds, sharing INT^2^GRATE data across broad clinical settings and diverse patient populations will help measure the efficacy and utility of this germline VUS assessment tool in patients from different population backgrounds.

The INT^2^GRATE decision tree is designed to use well-established clinical criteria, the combination of which favors a conservative approach to assessing VUS. All scenarios that lead to the INT^2^GRATE POSITIVE category are those that are clinically present when a germline variant is pathogenic. It is noteworthy that INT^2^GRATE is not intended to re-classify VUS or change the ACMG assessment criteria. Instead, it is designed to serve as a companion tool to help clinical professionals to efficiently collect and assess tumor-derived and clinical genetic evidence for each VUS. Selected clinically reported but uncommon scenarios were also included in the decision tree, but these criteria render the designation of INT^2^GRATE NEUTRAL (Methods under MMR IHC Pattern and Rationale). The comments explaining the combination of evidence in these scenarios are intended to be useful to those professionals who may not have access to readily available data from large testing centers. Lastly, data sharing is a main goal of INT^2^GRATE. Currently, there is no platform that enables systematic collection and data sharing of integrated tumor-derived and clinical genetics evidence for VUS. Sharing both tumor and germline variant-level information will help disseminate the clinical evidence related to VUS.

In the assessment of tumor profiles, several conditions were not included in the conservative INT^2^GRATE decision tree. Among *BRAF* alterations, INT^2^GRATE assesses only *BRAF* p.V600E (not other alterations in *BRAF*), as it is the most common and well-characterized alteration of *BRAF* in MMRd CRC (32, 70). Scenarios with the absence of a somatic second hit were excluded from the design. Indeed, we report 15 LS control patients with documented pathogenic germline variants that lacked a somatic inactivating variant in the MMR gene of interest, despite loss of expression of the corresponding MMR proteins by IHC. Given the various reasons for the absence of bi-allelic inactivating of MMR genes [e.g., assay limitations, epigenetic or complex structural alterations, technical issues, insufficient coverage, low tumor purity, or poor mapping quality particularly for *PMS2*, or age-related LOH contribution typically observed in younger patients (13)], these scenarios were excluded from the INT^2^GRATE POSITIVE criteria.

The current application of INT^2^GRATE in LS-associated cancers is limited to CRC and EC. These two are the most common cancers (excluding skin cancer) in LS, accounting for ~39% and ~10% of all cancers diagnosed in a large prospective LS cohort, respectively (71). Most laboratories in the US employ universal MMR/MSI testing for CRC and EC, but not for other LS-associated tumors. As somatic MMR testing is incorporated into oncology practice for all tumors, the algorithm may be expanded to include other LS-associated cancers and potentially even cancers not known to be associated with LS.

The application of the PREMM5 risk model or LS clinical criteria may present an ascertainment bias in selecting VUS that meet the eligibility criteria to be evaluated by INT^2^GRATE. In our positive control groups, five LS patients with pathogenic variants in *PMS2* or *MSH6* were excluded from the assessment based on the PREMM5 criterion alone. *MLH1* and *MSH2* have the highest penetrance for cancer of the MMR genes, approaching cumulative risks of 70-80% for all cancers, whereas penetrance of cancer in males with *MSH6* (~40%) and both males and females with *PMS2* is much lower (~35%) (71). PREMM5 considers the lower penetrance of *PMS2* and *MSH6* but is likely affected by some selection bias due to mild phenotype. We, therefore, expect that some VUS in *PMS2* and *MSH6* may not reach the necessary threshold to be assessed by INT^2^GRATE, especially in younger individuals without the manifestation of LS. A study of a larger set of MMR variants assessed by INT^2^GRATE may delineate the utility of INT^2^GRATE in different MMR genes.

In conclusion, INT^2^GRATE provides a platform to systematically collect relevant data in LS, to comprehensively assess the role of germline VUS using well-established tumor and clinical evidence, and to share both the collated tumor and germline data in publicly available variant databases. As both tumor and germline testing becomes more widely available in clinical settings, sharing integrated tumor and germline genomic findings is essential for translating genomic testing results into clinical knowledge. Sharing and accessing variant-level information using INT^2^GRATE will help mitigate the challenges in genomic oncology imposed by VUS.

## Data availability statement

The datasets presented in this article are not readily available because of ethical and privacy restrictions. Requests to access the datasets should be directed to the corresponding author.

## Ethics statement

The studies involving humans were approved by Brigham and Women’s Hospital (Protocol #: 2004P000062) and DFCI/BWH Pop Sci: Cancer Genetics (Protocol # 22-495). The studies were conducted in accordance with the local legislation and institutional requirements. The participants provided their written informed consent to participate in this study.

## Author contributions

RI: Data curation, Investigation, Methodology, Validation, Writing – original draft, Writing – review & editing. AC: Data curation, Investigation, Methodology, Validation, Writing – original draft, Writing – review & editing. MW: Data curation, Formal analysis, Investigation, Methodology, Validation, Visualization, Writing – review & editing. AS: Data curation, Investigation, Methodology, Writing – review & editing. DK: Data curation, Investigation, Methodology, Writing – review & editing. CH: Data curation, Formal analysis, Writing – review & editing. BU: Data curation, Formal analysis, Writing – review & editing. MM: Data curation, Formal analysis, Writing – review & editing. RB: Data curation, Writing – review & editing. DM: Data curation, Methodology, Writing – review & editing. LS: Writing – review & editing. MR: Data curation, Writing – original draft, Writing – review & editing. MY: Investigation, Writing – review & editing. HR: Investigation, Writing – review & editing. JG: Investigation, Writing – review & editing. AG: Conceptualization, Data curation, Formal analysis, Investigation, Methodology, Project administration, Resources, Supervision, Validation, Visualization, Writing – original draft, Writing – review & editing.
