# Supplementary material for: Development and evaluation of INT2GRATE: a platform for comprehensive assessment of the role of germline variants informed by tumor signature profile in Lynch syndrome
Source: Front Oncol. 2024 Jan 24;13:1284690. doi: 10.3389/fonc.2023.1284690 (PMC10854004; doi:10.3389/fonc.2023.1284690)
Supplement: Supplementary file 7 [file DataSheet_1.docx]

**SUPPLEMENTARY LABORATORY METHODS**

**Tumor Genetic Testing**

Tumor testing was performed by OncoPanel test for single nucleotide variants (SNV/Indel), copy number variants (CNV), structural variants (SV), and Mismatch Repair (MMR)/Microsatellite Instability (MSI) status, at the Pathology Department at Brigham and Women’s Hospital (BWH, Boston, MA, USA), as described previously [1].

OncoPanel SNV/indel Analysis

Tumor samples were analyzed with MuTect and GATK Indelocator (Broad Institute, Cambridge, MA, USA) to identify somatic SNVs and indels, respectively, as previously described [2, 3]. The OncoPanel pipeline filtered variants based upon several criteria, including presence in a panel of normal samples, those found in the Exome Sequencing Project (ESP) and/or gnomAD databases with the allele frequency of >0.1% in any sub-population, or detected in the plate normal control that is run with each assay. Any variant filtered by those criteria that are present in the COSMIC database (COSMIC, Wellcome Sanger, London, UK) at least twice were subsequently rescued. Non-filtered and rescued variants were presented for technical review in a web-based interface (OncoPanel NGS.Rev), which linked to a snapshot from the Integrated Genome Viewer (IGV) for each variant. Each variant was annotated with the gene, genome coordinates, reference and alternate alleles, coverage, allele fraction, cDNA, and protein change. In somatic OncoPanel validation, the limit of detection was determined to be 50X coverage and 10% variant allele fraction. Variants called with lower coverage and/or allele fraction, or with less than five unique reads of support were excluded from the analysis.

OncoPanel CNV Analysis

RobustCNV was developed at Dana-Farber Cancer Institute (DFCI, Boston, MA, USA) to detect somatic CNVs, as described previously [2, 3]. Each genomic segment baited in OncoPanel was normalized against the panel of normal samples, and the Log2 ratios were plotted for visualization in NGS.Rev. Segments with a Log2 ratio on the zero line were considered neutral. The overall landscape of a sample's copy number status was visible in the "all chromosome" view, and each individual chromosome was manually reviewed for chromosome-level, arm-level, and/or focal gains or losses. Low amplifications, high amplifications, one copy deletions, or two copy deletions may be recorded in technical review. Typically, low amplifications were called at a Log2 ratio ≥ 0.43 and losses at a Log2 ratio ≤ -0.32.

OncoPanel SV Analysis

BreaKmer was developed at Dana-Farber Cancer Institute (DFCI, Boston, MA, USA) to detect somatic chromosomal rearrangements, large indels, and inversions, as described previously [2-4]. The tool identifies sequence fragments that map to non-contiguous regions of the reference sequence. In NGS.Rev, SV fragments were presented with the gene(s) involved, genome coordinates (“breakpoints”) of the variants detected, and an IGV snapshot for visual confirmation. The coverage at each of the detected breakpoints was provided with the numbers and types of reads supporting the variant, which included split reads (a single read that maps to two regions of the genome) and discordant read pairs (the paired ends of a sequenced read map to different genomic locations). SV calls in repetitive regions of the genome were excluded from the analysis. Variants with support ≤ 2% (total split and discordant reads/total coverage across the detected breakpoints) were closely reviewed to confirm the variants were unique to the sample (i.e., not identified in unrelated patients or the normal control) and passed through to pathologist review.

OncoPanel MMR)/MSI Analysis

MMR status is calculated for every OncoPanel somatic sample based upon the number of homopolymer indels detected in non-filtered variants. A homopolymer indels is a one basepair insertion or deletion that occurs adjacent to a homopolymer that is ≥ 3 basepairs. The number of homopolymer indels detected in each sample was divided by the size of the exonic target regions in OncoPanel, which is 1.315 megabases (MB). Samples were considered MMR-deficient/MSI-high with a value greater than one.

**OncoPanel Data Query**

Using a custom query, all OncoPanel cases that have reported variants in any MMR genes were collected for analysis. Tumor microsatellite instability (MSI) status, tumor burden, and tumor type were also collected in this target pull. A secondary data query was performed to collect, this time, all reported variants in OncoPanel’s 447 genes for cases collected in the first target pull. This query enabled assessment of other variants beyond MMR genes (e.g., *BRAF*) in MMR positive cases.

**Paired Tumor-Normal Testing**

Paired tumor-normal testing was performed by TumorNext-Lynch in a commercial laboratory (Ambry Genetics, CA, USA) as described previously [5]. In this assay, DNA is obtained from blood or saliva representing germline and from tumor in paraffin-embedded specimens (minimum 20% neoplastic cellularity). Sequences for *MLH1, MSH2, MSH6, PMS2*, and *EPCAM* are enriched using bait-capture methodology followed by PCR and next-generation sequencing. Data is analyzed in a paired manner for both normal and tumor DNA to discriminate variants of somatic versus germline origin. Only *EPCAM* 3’ deletions are reported. Specific thresholds for satisfactory analysis include 20x depth for germline, 100x for tumor. Special testing for complex alterations (e.g., *MSH2* exon 1-7 inversion, *PMS2* pseudogene homology regions) may be used.

**Germline Testing**

Germline only testing was performed in a commercial laboratory (Invitae, San Francisco, CA, USA) according to previously published articles and protocols [1]. Briefly, DNA samples from peripheral blood were enriched for targeted regions using a hybridization-based protocol. Sequencing was performed using the Illumina NovaSeq 6000 platform (Illumina, San Diego, CA, USA). Using multi-gene panels, the analysis was performed on coding sequences as well as 10 bp flanking the intronic sequence (20 bp for BRCA1and BRCA2 genes) of target genes. Copy number alterations in exonic regions were processed by analysis of the read depth for each target sequence, mean read depth and read-depth distribution obtained from parameters in validation experiments.

**Immunohistochemistry (IHC)**

For cases stained at BWH, IHC staining for MMR proteins was performed as previously described [1]. Briefly, slides with formalin-fixed, paraffin-embedded tissue sections were deparaffinized, rehydrated, subjected to antigen retrieval, and incubated with the primary antibodies targeting MLH1 (1:300, clone G1680728; MLH1-L-CE, Leica), PMS2 (1:300, clone MRQ-28; 288m-16, Cell Marque), MSH2 (1:400, clone FE11; NA27, Oncogene), or MSH6 (1:800, clone PU29; NCL-L-MSH6, Leica). Secondary detection was achieved using the Leica Novolink detection system.

**References**

1. Schwartz, A., et al., *An integrated somatic and germline approach to aid interpretation of germline variants of uncertain significance in cancer susceptibility genes.* Front Oncol, 2022. **12**: p. 942741.

2. Manning, D.K., et al., *Assessment of genomic alterations in non-syndromic von Hippel-Lindau: Insight from integrating somatic and germline next generation sequencing genomic data.* Data Brief, 2021. **39**: p. 107653.

3. Rana, H.Q., et al., *Pathogenicity of VHL variants in families with non-syndromic von Hippel-Lindau phenotypes: An integrated evaluation of germline and somatic genomic results.* Eur J Med Genet, 2021. **64**(12): p. 104359.

4. Abo, R.P., et al., *BreaKmer: detection of structural variation in targeted massively parallel sequencing data using kmers.* Nucleic Acids Res, 2015. **43**(3): p. e19.

5. Dixon, K., et al., *Integrating Tumor Sequencing Into Clinical Practice for Patients With Mismatch Repair-Deficient Lynch Syndrome Spectrum Cancers.* Clin Transl Gastroenterol, 2021. **12**(8): p. e00397.
